# miRNA Signature in NAFLD: A Turning Point for a Non-Invasive Diagnosis

**DOI:** 10.3390/ijms19123966

**Published:** 2018-12-10

**Authors:** Paola Dongiovanni, Marica Meroni, Miriam Longo, Silvia Fargion, Anna Ludovica Fracanzani

**Affiliations:** 1 General Medicine and Metabolic Diseases, Fondazione IRCCS Ca’ Granda Ospedale Maggiore Policlinico, Milano 20122, Italy; maricameroni11@gmail.com (M.M.); longo.miriam92@gmail.com (M.L.); silvia.fargion@unimi.it (S.F.); anna.fracanzani@unimi.it (A.L.F.); 2 Department of Pathophysiology and Transplantation, Università degli Studi di Milano, Milano 20122, Italy

**Keywords:** non-alcoholic liver disease, NASH, fibrosis, HCC, PNPLA3, TM6SF2, MBOAT7, microRNAs, epigenetics, intestinal permeability

## Abstract

Nonalcoholic fatty liver disease (NAFLD) defines a wide pathological spectrum ranging from simple steatosis to nonalcoholic steatohepatitis (NASH) which may predispose to liver cirrhosis and hepatocellular carcinoma. It represents the leading cause of hepatic damage worldwide. Diagnosis of NASH still requires liver biopsy but due to the high prevalence of NAFLD, this procedure, which is invasive, is not practicable for mass screening. Thus, it is crucial to non-invasively identify NAFLD patients at higher risk of progression to NASH and fibrosis. It has been demonstrated that hepatic fat content and progressive liver damage have a strong heritable component. Therefore, genetic variants associated with NAFLD have been proposed as non-invasive markers to be used in clinical practice. However, genetic variability is not completely explained by these common variants and it is possible that many of the phenotypic differences result from gene-environment interactions. Indeed, NAFLD development and progression is also modulated by epigenetic factors, in particular microRNAs (miRNAs), which control at post-transcriptional level many complementary target mRNAs and whose dysregulation has been shown to have high prognostic and predictive value in NAFLD. The premise of the current review is to discuss the role of miRNAs as pathogenic factors, risk predictors and therapeutic targets in NAFLD.

## 1. Introduction

Nonalcoholic fatty liver disease (NAFLD) ranges from simple steatosis, defined by fat accumulation exceeding 5% of liver weight in the absence of alcohol abuse, to the progressive form namely nonalcoholic steatohepatitis (NASH), which is characterized by lobular inflammation and hepatocellular ballooning, and to hepatic fibrosis [1]. NASH may progress to cirrhosis and, in a small percentage of patients, to hepatocellular carcinoma (HCC) [2,3]. NAFLD is becoming a leading cause of liver damage worldwide, affecting between 20% to 40% of the adult population [4].

Pathogenesis of NAFLD is closely intertwined with excessive adiposity, insulin resistance (IR) and dyslipidemia [5]. Dietary factors such as excessive caloric intake, fructose and physical inactivity represent other risk factors for this condition [6]. Moreover, NAFLD has a strong genetic component, and variants in proteins regulating hepatocellular lipid handling, including Patatin-like Phospholipase Domain-containing 3 (*PNPLA3*), Transmembrane 6 Superfamily Member 2 (*TM6SF2*), Membrane Bound *O*-acyltransferase Domain-containing 7 (*MBOAT7*), predispose to the development and progression to NASH and fibrosis [7]. However, less than 10% of genetic variability is explained by these common variants and it is possible that many of the phenotypic differences result from gene-environment interactions, thus explaining the impossibility, till now, to define NAFLD heterogeneity at genomic level [8]. Epigenetics, a hereditable but reversible phenomenon that affects gene expression without modifying DNA sequence, provides a new perspective on NAFLD pathogenesis and management [9]. The epigenetic modulation of gene expression may occur in response to environmental cues as (1) modification of DNA nucleotides (e.g., methylation); (2) modifications of histones that determine DNA packing and accessibility; and (3) regulation of transcription by altering mRNA stability and activity due to specific binding of small RNA molecules such as microRNAs (miRNAs).

Epigenetic modifiers in NAFLD can represent novel molecular indicators which can determine not only the early risk assessment but also the disease progression and prognosis. Indeed, to date it is not possible to predict NAFLD outcome through the routinely used blood and tissue biomarkers due to their limited prognostic efficacy, tissue specificity and sensitivity. Liver biopsy remains the gold standard procedure for diagnosis of NASH. However, it is invasive, potentially dangerous and is generally performed when disease has progressed to clinically significant stages. Furthermore, due to the high prevalence of NAFLD, liver biopsy is not practical for mass screening, not being feasible in the 30% of the population affected by NAFLD, including children and elderly patients, and for follow-up and treatment monitoring. In this context, there is a growing need to identify novel and trustworthy biomarkers, attempting to distinguish NASH from simple steatosis and to define severity of fibrosis. Thus, circulating miRNAs have become appealing candidate molecules for diagnosis and staging of liver diseases.

miRNAs control at post-transcriptional level many complementary mRNA targets and their dysregulation has been shown to have high prognostic and predictive value in a broad spectrum of rodents and human pathological liver conditions, including NAFLD [10]. Moreover, miRNAs expression in human diseases appears to be tissue-specific. This would allow to identify miRNAs secreted from the liver and to correlate them with the severity of the disease. In addition, miRNAs possess high stability in formalin-fixed tissues and even in stored plasma samples, allowing their use also in retrospective studies. Finally, circulating miRNAs have a key role as signaling molecules because they are involved in cell-to-cell communication, as it occurs between hepatocytes and hepatic stellate cells (HSCs) whose cross-talk may result in fibrogenesis and liver damage progression.

The role of miRNAs as pathogenic factors and their use as diagnostic/prognostic biomarkers in NAFLD is the subject of this review, shedding light on the potential of miRNAs for the design of novel therapeutic drugs.

## 2. miRNAs

MicroRNAs (miRNAs) are short non-protein coding, single-strands RNAs of 19–22 nucleotides, that have a pivotal role in the regulation of gene expression [11]. miRNAs can target mRNAs through complementary base-pairing, thereby leading to the post-transcriptional repression of targeted protein-coding genes [11,12,13]. To date, there are more than 2000 known miRNAs encoded in various intergenic, intronic or exonic sequences and it is estimated that miRNAs may directly target up to 60% of all human genes [14]. Firstly transcribed as primary miRNAs (pri-miRNAs), these molecules are further processed in two steps, within the nucleus and then in the cytoplasm, to form mature miRNAs which exert either the silencing of target mRNAs or the repression of protein synthesis [11]. The targets of miRNAs can be multiple genes (multi-functionality) or multiple miRNAs can target single gene (redundancy) [15] suggesting that miRNAs have an extensive regulatory capacity and a profound impact on health and disease [11]. Inherited variations that affect miRNAs-mRNA complementarity may influence the repressive role of miRNAs, providing a possible explanation for individual variability of transcriptome, through genetic and epigenetic interactions [16]. In addition, miRNA-coding genes are exposed to regulatory mechanisms including DNA methylation and RNA editing, similarly to protein-coding genes, leading to alteration in miRNA stability and to their degradation [15].

miRNAs have been revealed in serum, plasma, saliva and urine and their association with proteins, lipids and lipoproteins make them more stable in the circulation. Usually miRNAs are found in complex with proteins, mainly Argonaute-2 (AGO2) [17] or packaged in extracellular vesicles, mostly exosomes [18] where they are protected from RNase degradation. The release of circulating miRNAs can occur via passive process during cell death or via active release of microvesicles from cells [19]. For example, it has been demonstrated that the liver-specific miR-122 is found mostly in the exosome-rich fraction in alcoholic and nonalcoholic liver injury, while it is predominant in the protein-rich fraction in acetaminophen-induced toxic liver injury [20]. This suggests that the distribution of miRNAs in the different compartments, protein versus vesicles, could provide additional information regarding the pathogenesis and the identification of a specific miRNA pattern as biomarkers of liver diseases.

### Exosomal miRNAs

Exosomes are small vesicles (30–90 nm) which are released from many cellular types into the extracellular space. Exosomes are considered the newest family member of “bioactive vesicles”. They reflect their tissue or cell of origin by the presence of specific surface proteins and play a key role in cell-to-cell communications (neighboring or distant cells), thus influencing the phenotype of recipient cells. As above mentioned, some miRNAs preferentially are packaged and released in exosomes (exomiRs) [21,22], suggesting that parent cells have mechanism to transfer these miRNAs into exosomes. Moreover, exosomal miRNA expression is altered under pathological conditions [23]. In addition, with the discovery that exosomal miRNAs can function as ligand of Toll-like receptors (TLRs) thus activating immune cells, a new interest in exosome study has been opened up [24]. 

Diverse methods are used for exosome isolation and this surely represents a limitation for their use since different purification strategies may affect exosomal miRNA content [25]. Furthermore, the large number of exomiRs may regulate different signaling pathways thus producing addictive effects in recipient cells. Finally, it is difficult to measure the concentration of miRNAs carried by an exosome. It is worth adding that the combination of exosomes with RNA interfere technology (RNAi) is a promising method for gene therapy [26].

## 3. miRNA Signature in NAFLD

miRNAs may regulate a wide spectrum of biological processes and metabolic homeostasis, including lipid synthesis, fatty acids and glucose catabolism, inflammation, proliferation, apoptosis and necrosis, which have been known to be epigenetically deregulated in NAFLD [27]. Therefore, alteration of miRNA expression in response to genetic/epigenetic factors or environmental conditions may contribute to steatosis onset and NAFLD progression [28]. The enhanced cell death, that typically occurs in NASH during ballooning degeneration, determines the release of several miRNAs into the circulation. Therefore, circulating miRNAs have a role of physiological significance in the biology of NAFLD and they mirror the histological features and the molecular events occurring in NAFLD [29]. Thus, they could be exploited as attractive candidate biomarkers for an accurate profiling of the different stages of the liver injury, enabling the early diagnosis and the clinical monitoring of the disease progression. Altered hepatic miRNA profile has been described in NAFLD and NASH both in humans and in experimental models [10,30,31,32]. Several differentially expressed miRNAs have been reported in plasma samples of NAFLD patients. Pirola et al. explored the circulating miRNA signature associated with NAFLD revealing that, among 84 miRNAs analyzed, miR-122, miR-192, miR-19a/b, miR-125b and miR-375 were up-regulated in simple steatosis and, in particular, miR-122, miR-192 and miR-375 were even more dramatically enhanced in NASH [29], potentially distinguishing NASH from simple steatosis.

### 3.1. miR-122

miR-122 is the most abundant miRNA in human liver [33], representing more than 70% of the total liver miRNA pool. During hepatocytes maturation, miR-122 stimulates the expression of 24 hepatocytes-specific genes, including hepatocyte nuclear factor 6 (*HNF6*) [34] and in liver regeneration it has been reported to regulate hepatocytes proliferation and differentiation, recapitulating the developmental processes [35]. It also interacts with numerous target genes involved in lipid and cholesterol metabolism [36].

Serum miR-122 correlates with alanine aminotransferase (ALT) levels and liver fibrosis in NAFLD patients. It also performs slightly better in the diagnosis of NAFLD severity than classical liver disease markers, including aspartate aminotransferase (AST), ALT and plasma caspase generated cytokeratin-18 (CK18) fragments [37]. Moreover, serum miR-122 was also positively correlated with serum concentrations of CK18 [29] which has been proposed as a marker of NASH and disease severity.

Several studies provided evidence that chronic liver diseases derived from of different etiologies are frequently correlated with increased miR-122 release in the circulation, thus representing a marker of liver damage in general. However, Pirola et al. revealed that serum miR-122 was up-regulated 7.2-fold in NASH patients compared to healthy subjects and 3.1-fold compared to simple steatosis, proposing it as an extra-hepatic fingerprint of NASH and as a hallmark of ballooning [29]. Thus, a modulation of miR-122 expression may recapitulate many of the changes observed in the natural history of NAFLD. However, an inverse correlation between hepatic and serum miR-122 levels has been detected [10]. It has been shown that miR-122 was 10-fold down-regulated in the liver of NASH patients compared to simple steatosis and was preferentially expressed at the edge of fatty-laden hepatocytes, suggesting that the lower expression of miR-122 in liver could be a consequence of a dynamic regulation of the rate of miRNA release into the circulation. In addition, the increased levels of serum miR-122 were positively correlated with the severity of NAFLD in murine models, even in absence of ALT elevation [38,39]. On the contrary, mice deficient for miR-122 rapidly developed NASH due to heightened lipogenesis, impaired lipid secretion, up-regulation of Interleukin 6 (*IL-6*), Tumor necrosis factor-alpha (*TNF-α*), C-C Motif Chemokine Ligand 2 (*CCL2*) and the recruitment of immune cells. Reduction of miR-122 is also involved in the up-regulation of fibrotic pathways by inducing Hypoxia-Inducible Factor 1-α (*HIF1α*) and Mitogen-Activated Protein Kinase 1 (*MAPK1*) and in HCC development. These data suggest that miR-122 has a role in NAFLD progression [40,41]. miR-122 deleted mice displayed also lower serum triglyceride (TG) and cholesterol levels and exhibited hepatic lipids accumulation. Consistently, mice treated with antagomirs which are nucleotides directed against miR-122, displayed 30% decrease of plasma cholesterol and enhanced β-oxidation [42]. miR-122 silencing induced the up-regulation of genes involved in lipids catabolism and favored lipogenesis in cellular models [10]. Opposite results were obtained after miR-122 over-expression [43] pointing out miR-122 as a promising therapeutic target in the treatment of hypercholesterolemia and other dyslipidemias [44].

Enhanced circulating levels of miR-122 have also been detected in patients with other liver diseases, such as viral hepatitis B and C (HBV, HCV) as well as in alcohol-and drug-induced liver injuries [45,46,47,48]. As in alcoholic and nonalcoholic liver injury, hepatic miR-122 was down-regulated also in patients with chronic HBV infection and inversely correlated with hepatic necroinflammation and viral load. By targeting cyclin G1, miR-122 prevents cyclin G1-p53 interaction thus hampering p53-mediated inhibition of HBV replication [49]. Conversely, miR-122 expression is paradoxically increased during HCV infection to prompt viral replication [50], possibly discerning these two viral infections [51]. Also, alcohol consumption has been proven to be involved in HCV replication. Indeed, ethanol-induced higher levels of miR-122 which, in turn, targets several disease modifiers in HCV infection further contributing to viral replication [52,53].

### 3.2. miR-192

miR-192 is a pro-fibrogenic molecule induced by transforming growth factor β 1 (*TGFβ1*), that is involved in fibrosis development and in the activation of TGFβ/SMAD signaling. miR-192 was found to be mainly expressed in quiescent HSCs, favoring the inhibition of HSCs activation, proliferation and migration [54].

As previously described for miR-122, also miR-192 was increased in NASH serum compared with steatosis and down-regulated in NASH liver, both in human and animal models suggesting that these miRNAs are released from hepatocytes during pathophysiological states associated with cell membrane impairment [37] and they could be proposed a possible NASH biomarkers.

### 3.3. miR-375

miR-375 is considered to be a key regulator of glucose homeostasis and it is essential for β-cells expansion in response to increasing insulin demand in insulin resistant conditions. It has been found significantly up-regulated in NASH patients compared to simple steatosis [29]. It has been recently described that miR-375 expression was increased in serum of mice fed high fat diet (HFD) compared to healthy controls. Moreover, inhibition of miR-375 up-regulated the expression of adiponectin, inhibited lipid accumulation and down-regulated both the level of leptin and inflammatory cytokines including *TNF-α* and *IL-6* in human hepatocellular carcinoma cells (HepG2) treated with fatty acids. In addition, it has been demonstrated that adiponectin receptor 2 (AdipoR2) was a target of miR-375 which directly binds to its 3’UTR. These findings highlighted the potential use of miR-375 as a novel therapeutic target in NAFLD [55]. miR-375 seems to be involved also in HCC. Previous studies have shown that miR-375 mimic inhibits autophagy in HCC through targeting the Autophagy-related gene 7 (*ATG7*) [56]. In addition, miR-375 suppresses malignant phenotypes of HCC by targeting Astrocyte elevated gene-1 (*AEG-1*) and Yes-associated protein 1 (*YAP1*) which mediate steatosis and hepatic proliferation/differentiation respectively [57,58].

## 4. miRNA Signature in Progressive NAFLD

In addition to the aforementioned miRNAs, which better discriminate simple steatosis from NASH, several other miRNAs have been identified to exert a central role in the development of steatosis and its progression to NASH, fibrosis and HCC. Cermelli et al. revealed the presence of increased serum concentrations of miR-122, miR-34a and miR-16 in NAFLD patients compared to healthy controls and a correlation of their expression with liver enzymes, inflammation and fibrosis stage, suggesting that they may represent novel non-invasive biomarkers of progression from steatosis to NASH [45]. Yamada et al. found enhanced serum abundance of miR-122, miR-21, miR-34a and miR-451 in a cohort of NAFLD-diagnosed patients compared to controls, paralleling the steatosis grade [39]. Tan et al. proposed miR-1290, miR-27b-3p and miR-192-5p as a panel of high diagnostic accuracy for NAFLD, regardless the NAFLD activity score (NAS). This panel has a significant clinical value in NAFLD diagnosis, since it is more sensitive and specific than ALT and Fibrosis 4 (FIB-4) score [59]. Guo et al. reported that NAFLD severity is associated with a specific pattern of altered hepatic miRNA expression that may drive the hallmark of this disorder, altered lipid and carbohydrate metabolism. Specifically, the authors indicated miR-301a-3p, miR-34a-5p and miR-375 as potential markers of NAFLD severity [60]. Over the past years, evidence has emerged that miRNAs which are involved in the regulation of hepatic cholesterol and lipid metabolism (i.e., miR-122, miR-33a/b, miR-29 and others) may further mediate the development of metabolic disturbance, atherosclerosis and cardiovascular disease, confirming NAFLD as a systemic disorder [61]. More recently, Blaya et al. revealed that miR-155 expression is altered in both liver tissue and circulating inflammatory cells during liver injury, regulating inflammatory cells recruitment and liver damage. Thus, modulation of miR-155 has been proposed to be a potential strategy to damp liver injury [62]. Finally, Zhu et al. identified 17 miRNAs, among which miR-144-3p, miR-99a-3p, miR-200b-3p, miR-200b-5p, as novel potential biomarkers of NAFLD [63].

### 4.1. miR-33a and miR-33b

miR-33a and miR33b are co-transcribed with the Sterol regulatory element-binding protein 1 (*SREBP1*) and *SREBP2* [64], key regulators of de novo lipogenesis and cholesterol biosynthesis, localizing in the intronic regions of SREBP genes. miR-33a and miR-33b are positive regulators of these two transcription factors and both target the α-subunit of the AMP-activated protein kinase (*AMPK*), inhibiting its expression [65,66]. Moreover, they also play a key role in post-transcriptional repression of ATP-binding cassette transporter sub-family A member 1 (ABCA1), which is essential for cholesterol binding to Apolipoprotein A1 (APOA1) during high density lipoprotein (HDL) formation [67]. For this reason, they contribute to the modulation of fatty acids metabolism, cholesterol synthesis and insulin signaling pathways. Therefore, the inhibition of these miRNAs enhanced insulin sensitivity, fatty acids oxidation and circulating levels of HDL as well as reduced lipid accumulation in arterial plaques, raising the possibility to use them as therapeutic targets in the management of metabolic syndrome, atherosclerosis and NAFLD [68]. Likewise, miR-33a was highly expressed in activated HSCs due to the modulation of phosphatidylinositol-3-kinase/protein kinase B (PI3k/PKB) pathway and correlated with *TGFβ*-induced expression of type I collagen (*Col1A1*) and α-smooth muscle actin (*αSMA*), suggesting its involvement in fibrosis development [69,70].

### 4.2. miR-34a

miR-34a is highly expressed in patients with type 2 diabetes mellitus (T2DM), steatosis, NASH and in experimental models of NAFLD [71,72]. It was positively correlated with very low-density lipoprotein (VLDL), TG and serum ALT. miR-34a is the most characterized regulator of Sirtuin 1 (*SIRT1*), a NAD-dependent deacetylase [45]. miR-34a is a highly lipid responsive miRNA in the liver, modulating oxidative stress and metabolism, and it has been reported that it regulates apoptosis, DNA damage and telomere attrition in cardiac senescence and ischemic diseases [73]. Silencing of miR-34a restored the expression of *SIRT1* and Peroxisome proliferator-activated receptor α (*PPARα*), resulting in activation of *PPARα* downstream genes, such as *AMPK* and Hydroxymethylglutaryl-CoA Reductase (*HMGCR*), thus ameliorating steatosis [74]. Conversely, induction of miR-34a is related to Hepatocyte nuclear factor 4α (*HNF4α*) inhibition in NAFLD patients, inflammation through Nuclear Factor Kappa-Light-Chain-Enhancer of Activated B Cells (NF-κB) activation and hepatocellular apoptosis [72]. Xu et al. demonstrated that miR-34a inhibited VLDL secretion from the liver, promoting steatosis onset through the interaction with HNF4α in NASH patients and in HFD-fed mice [75]. In addition, the over-expression of miR-34a in vitro exacerbated free fatty acids (FFA)-induced oxidative stress and apoptosis [76]. Notably, miR-34a has been also recognized to act on the repression of β-Klotho, a co-receptor of the Fibroblast growth factor 19 (FGF19), implicated in glucose metabolism during post-prandial response in physiological states. Indeed, inhibition of miR-34a in *ob/ob* mice ameliorates obesity and restores β-Klotho/FGF19 signaling [28,77].

### 4.3. miR-451

Hepatic lipid accumulation and inflammation usually occur together. There is a growing evidence supporting the role of innate immunity in NASH. In particular, TLR signaling induces the expression of a variety of miRNAs, as well as several miRNAs can modulate TLRs [78]. miR-451 is responsive to fatty acids and a reduced hepatic expression of miR-451 is detected in human NASH, in HepG2 cells treated with palmitic acids and in mice fed HFD [79]. miR-451 could regulate dendritic cell cytokines and suppress chemotaxis. Down-regulation of miR-451 led to increased activation of NF-κB and to enhanced secretion of IL-8 and TNF-α [79]. Conversely, the over-expression of miR-451 decreased inflammatory cytokines release [79]. These evidences highlight new insights into the negative regulation of miR-451 in fatty acid-induced inflammation, pointing out its potential therapeutic applications [15].

### 4.4. miR-155

One of the miRNAs that could regulate Kupffer cells (KCs) is miR-155. It is involved in inflammatory processes that control innate and adaptive immunity in both alcoholic and nonalcoholic fatty liver disease [80,81]. It was found highly expressed in hepatocytes and in KCs, isolated from mice fed Methionine Choline-deficient diet (MCD), where it contributes to Lipopolysaccharides (LPS) sensitization and TNF-α production [80,81]. In particular, miR-155 was up-regulated in response to LPS and enabled TNF-α mRNA stability and protein synthesis. By promoting the proinflammatory process through TNF-α, miR-155 may be further favor atherosclerotic plaque rupture [82]. Conversely, in isolated KCs the treatment with IL-10 or TGFβ suppressed miR-155 expression [83]. Consistently, in RAW264.7 macrophages, the immunosuppression of KCs/macrophages could be positively regulated by miR-155 inhibition. Indeed, in liver ischemia-reperfusion injury mice, miR-155 deficiency resulted in the development of M2 macrophages and produced a regulatory inflammatory response with higher levels of IL-10 and lower levels of TNF-α, IL-6 and IL-12p40 [84]. However, miR-155 knock-out mice failed to hamper liver inflammation although steatosis and fibrosis were reduced [81]. In fact, it plays also an important role in the initial hepatic lipid accumulation, targeting Liver X receptor alpha (*LXRα*) and modulating lipid metabolism [81].

miR-122, miR-192, miR-155, miR-34a are detectable in liver diseases from different etiologies [27]. However, it has been observed that they are more up-regulated in patients with NASH, possibly representing specific biomarkers for this pathological condition. To date, it is difficult to assign a miRNA profile, which is specific for NASH and further studies are surely required.

## 5. Role of miRNAs in Adipose Tissue and Gut Homeostasis

The excessive expansion of visceral adipose tissue (VAT) results in chronic low-grade inflammation and adipocytes along with macrophages secrete several mediators (referred to as adipokines), that are essential in the complex cross-talk between liver and adipose tissue which occurs in patients with metabolic syndrome [85]. Several adipokines have been reported to be related to changes in miRNA profile and differential miRNAs expression was found not only in the liver, but also in VAT of NAFLD patients [86]. In biopsy-proven NASH, the VAT expression of miR-7.1, miR-132, miR-150, miR-433, miR-28-3p, miR-511, miR-517a, miR-671 is statistically different compared to healthy subjects [86]. Likewise, in NASH patients, VAT altered gene expression of miRNAs processing enzymes (*Dicer1*, *Drosha*, *DGCR8*) was also reported. Thus, obesity results in a unique miRNA profile and different stages of NAFLD are related to diverse miRNA signatures. Some of those differentially expressed miRNAs correlate with body weight and metabolic parameters, affecting adipocytes differentiation, adipose tissue inflammation and hypothalamic regulation of energy homeostasis. For example, miR-125b levels were positively related to fasting glucose, TGs concentrations and to the body mass index (BMI) both in serum and in VAT [29,87].

It has also been described that miRNA-containing adipose tissue macrophages (ATM)-exosomes can modulate systemic insulin and glucose tolerance by directly affecting cellular insulin signaling. miR-155 contributes to the insulin resistant, glucose intolerant state conferred by obese ATM-Exos by repressing PPAR-γ, possibly in a coordinated way with other miRNAs. It could be speculated that ATM-Exos miRNAs represent a paracrine and endocrine signaling system, whereby proinflammatory and anti-inflammatory ATMs can influence metabolic events in distant tissues [88]. Chai et al. found that FFAs increase hepatic expression and secretion of miR-122, which regulates energy storage versus expenditure in liver and peripheral tissues. Strategies to reduce triglyceride levels, by increasing miR-122, might be developed for treatment of metabolic syndrome [89].

Additionally, gut microbiome changes influence obesity and the pathogenesis of NAFLD/NASH. Indeed, the liver is exposed to high concentration of gut-derived microbial components and a well characterized feature of NAFLD is represented by the increased intestinal permeability, resulting in enhanced steatosis and inflammation, due to the high influx of TLRs ligands [90]. miRNAs may modulate also the expression of genes involved in microbial recognition or vice versa they may be induced by microbial products. Indeed, several studies demonstrated that germ-free mice colonized with the microbiota from pathogen-free mice induced changes in host miRNAs, in response to microbiota colonization, suggesting that the intestinal bacteria participate also in the modulation of miRNAs, which could in turn regulate host gene expression. Mice carrying intestinal epithelial cell (IEC)-specific Dicer deficiency displayed higher levels of miR-122 which induces a degradation of occludins leading to an increased intestinal permeability [91]. Similarly, miR-375, miR-146a, miR-155, miR-29 and miR-10a were implicated in the alteration of intestinal environment. Particularly both miR-212 and miR-155 can down-regulate components of tight junctions including Zonula Occludens 1 (ZO-1) and up-regulate the levels of inducible Nitric oxide synthases (*iNOS*) resulting in intestinal barrier dysfunction [27]. In particular, miR-212 is highly expressed in intestine and the ethanol exposure could induce miR-212 expression in Caco2 cells. In HFD mice, the relative abundance of *Firmicutes* was negatively correlated with hepatic expression of miR-666 and miR-21. In contrast, the relative abundance of *B. acidifaciens* was positively correlated with miR-21 suggesting that gut microbiota may govern hepatic pathophysiology during metabolic alterations [92]. Moreover, miR-21 knock out mice displayed attenuated inflammation and tissue injury, improving the survival rate in dextran sulfate sodium (DSS)-induced fatal colitis [93].

## 6. miRNAs in Liver Fibrosis and HCC

Hepatic fibrosis is the most frequent outcome of chronic liver disease, characterized by deposition of extracellular matrix (ECM) and alteration of normal hepatic parenchyma. It has been described that the stage of fibrosis may be the main cause of vascular events and non-hepatic cancer, to which 50% of deaths are attributable [94]. Approximatively 15–30% cases of NASH may progress to hepatic fibrosis and encounter portal hypertension, cirrhosis development and eventually liver failure [95,96]. Furthermore, high percentage of patients with NASH develop HCC either in the presence or not of cirrhosis, being HCC the fifth most prevalent cancer and the third leading cause of cancer-related deaths worldwide [97].

The key event of hepatic fibrogenesis is represented by the activation of HSCs. They amount to ~8% of hepatic cell types, localized in the space of Disse and maintain a non-proliferative quiescent state until stimulation. Chronic hepatic injury of any etiology and inflammatory signals (i.e., TGFβ) could be the main cause of HSCs trans-differentiation into myofibroblast-like phenotype, also termed activated state. Activated HSCs are the primary source of scar tissue deposition and are featured by higher cell proliferation rate, loss of vitamin A-containing lipid droplets, induction of *αSMA* and drive fibrosis mainly by secreting ECM proteins and fibrillar collagens [98]. Likewise, repeated cycles of disruption and compensatory tissue regeneration due to sustained inflammation and hepatic damage may lead to hepatic carcinogenesis [97].

As highlighted, the study of epigenetic mechanisms is achieving considerable relevance to discover novel blood-based noninvasive biomarkers to monitor and manage NAFLD progression whereby treatment is still available.

As previously mentioned, Cermelli et al. observed that miR-122, miR-34a and miR-16 circulating levels were strongly correlated with liver enzymes, inflammation activity and fibrosis score [45]. Confirming these findings, in a retrospective study which considered 52 patients with fibrosis of various etiologies, miR-122 was negatively correlated with fibrosis stage and liver stiffness values [99]. Conversely, Celikbilek et al. showed that miR-181d, miR-99a, miR-197 and miR-146b levels were significantly reduced in sera of 20 histologically proven NAFLD compared to control group and miR-197 and miR-10b negatively correlated with inflammation. However, this study did not reveal changes in miR-122 and miR-34a serum expression in NASH patients and healthy controls [100]. Additionally, many other reports showed deregulated hepatic levels of miRNAs by comparing tumoral tissues to non-cancerous ones. Although analysis of miRNA signature in HCC samples embedded by bioinformatic tools, revealed several miRNA candidates and their target genes involved in liver tumorigenesis, these results do not always overlap, possibly due to different HCC etiologies, genetic background, sex, age but also biases from technique protocols or differences in patients’ samples [101].

Despite the clinical relevance of liver fibrosis and cancer, the mechanisms underlying hepatic fibrogenesis and carcinogenesis are still under definition thus constraining appropriate strategies for prevention and pharmacological therapies. In this contest, identification of specific miRNA panel related to liver disease severity seems to be a promising non-invasive approach for diagnosis, prognosis and preventive interventions [68].

### 6.1. miR-15 and miR-16

miR-15 and miR-16, both members of miR-15 family, also including miR-497, miR-195 and miR-322 [102], raised a great interest for their involvement in liver fibrosis and hepatocarcinogenesis. Higher circulating levels of miR-16 were observed in individuals affected by NAFLD compared to healthy controls and correlated with the severity of liver disease, supporting their use as biomarkers for NAFLD staging [45]. miR-16 dysregulation may promote liver fibrosis in HSCs by up-regulating guanine nucleotide-binding α-subunit 12 (Gα12), a key transductor of G-protein coupled receptors (GPCRs) network signaling. Gα12 interaction with ATG12-5 is a pro-fibrogenic signal which stimulates HSCs activation through autophagy-mediated breakthrough of lipid droplets [103,104].

In addition, Guo and coworkers revealed a set of 21 differential miRNA expression by comparing rat quiescent HSCs to activated ones. Among them, examination of the seed region in miR-15/16 sequence showed complementarity of six nucleotides with anti-apoptotic B-cell lymphoma-2 (*Bcl2*) factor at 3’UTR mRNA sequence [105]. Indeed, miR-15 and miR-16 down-regulated Bcl-2 at post-transcriptional level and their expression is reduced during HSCs activation. Primary rat HSCs derived by carbon tetrachloride (CCl_4_)-induced hepatic fibrosis model showed increasing Bcl-2 levels and lower expression of caspase-9 compared to normal controls, supporting their pro-apoptotic role in HSCs via targeting Bcl-2 and members of caspase signaling [105,106].

miR-15/16 are also involved in the regulation of cell cycle progression, proliferation and oncogenesis [106]. miR-15/16 clusters are well-known tumor suppressor, by inhibiting numerous oncogenes and their deregulation was also observed in multiple in vitro and in vivo models [107,108]. In MHCC97H, a high invasive subtype of HCC cell line, miR-15a expression was lower than in a less tumorigenic hepatocytes cell line (Huh7), suggesting its levels might be associated to more aggressive HCC phenotype [108]. In patients with HCC, miR-15a levels were reduced in cancerous hepatic tissue and they negatively correlated with lymph nodes metastasis and TNM classification [109]. Besides miR-15a, aberrant expression of miR-16 is also implicated in cancer development. HepG2 over-expressing miR-16 exhibited less proliferative, invasive and metastatic phenotype and these effects may be attributed to increased apoptotic-related proteins and diminished levels of metalloproteases. In addition, transient transfection of HepG2 cells with miR-16 inhibitor showed growing levels of PI3K and phosphorylated Akt, which are widely involved in numerous biology processes among which proliferation, migration, invasion and ECM formation [110].

### 6.2. miR-34

Members of miR-34 family gained great attention for their pleiotropic functions in regulation of proliferation, differentiation and programmed cell death. miR-34a is one of the most increased miRNAs during hepatic fibrogenesis in both animal models and human patients with different hepatopathies [10,111]. In rodents, miR-34a inhibits the acyl-CoA synthetase 1 long-chain family member 1(*Acsl-1*) enzymatic activity, which promotes lipid accumulation in hepatocytes and HSCs [112,113,114]. Silencing of miR-34a up-regulates *Acsl-1* and decreases the expression of *αSMA*, *Col1A1* and *desmin* as well as prompts lipogenesis in HSCs by up-taking fatty acids and contributing to maintain quiescent HSC phenotype [114]. In addition, the interaction of miR-34a and miR-34c with PPARγ, an anti-fibrotic factor, represents another mechanism of regulation of human HSCs activation [113]. In hepatocytes, miR-34a targets *SIRT1* and caspase 2 (*CASP2*), which commonly increase apoptosis susceptibility, also contribute to ECM remodeling via up-regulation of metalloproteinase 2 and 9 (*MMP2*, *MMP9*) [111,115].

miR-34a, apoptosis and acetylated-p53 increase with disease severity whereas SIRT1 expression is reduced in NAFLD patients. Throughout hepatic injury, increased miR-34a levels promote apoptosis by targeting *SIRT1*. Rats with CCl_4_-induced fibrosis showed reduced miR-34a levels and p53 acetylation in hepatocytes but not in HSCs upon SIRT1 activator exposure. Such findings support a cell-specific mechanism of action of miR-34 to promote hepatic fibrogenesis [111,115]. Ursodeoxycholic acid (UDCA) is a potent inhibitor of p53-dependent pathways. The p53/miR-34a/SIRT1 pro-apoptotic pathway is prevented by UDCA in primary rat hepatocytes even after miR-34a overexpression, a condition that mimics increased miR-34a levels in patients with severe NAFLD [72]. A possible explanation is that UDCA, by blocking p53 transactivation, inhibits mir-34a expression and on the other hand it seems that modulation of SIRT1 by UDCA depends almost exclusively on miR-34a. The inhibition of the p53/miR-34a/SIRT1 pathway prevented the miR-34a-mediated repression of SIRT1 and lead to reduced p53 acetylation and apoptosis, thus representing a novel therapeutic target for NAFLD progression.

miR-34a is also becoming highly studied in cancer although its role is still controversial [116,117]. miR-34a may affect hepatocytes proliferation and apoptosis. Hep3B and HepG2 cell lines over-expressing miR-34a showed higher cell death rate, as proven by increased expression of anti-apoptotic proteins (i.e., Bcl2), and inversely correlation of Histone deacetylase 1 (HDAC1) expression, which is currently considered as therapeutic target in malignancies. Comparison between cancerous and adjacent normal hepatic tissues of 60 patients with HCC confirmed that miR-34a levels were lower whereas *HDAC1* mRNA expression was markedly higher in tumor samples [118]. The anti-cancer effects of 0404, a novel DNA-damaging compound, were investigated in several human HCC cell lines and HCC xenograft mouse model. 0404 up-regulates miR-34a and increased p53 acetylation in both in vitro and HepG2-bearing nude mice, suggesting that its anti-tumoral activity on HCC was exerted via p53/miR-34a/SIRT1 pathway [119].

### 6.3. miR-21

miR-21 is one of the most up-regulated miRNAs in serum and hepatic tissues of individuals with fibrosing-NASH and HCC [120,121]. Rodrigues et al., for the first time, reported that miR-21 knock-out animals fed a fast food diet supplemented with obeticholic acid (OCA) displayed minimal steatosis, inflammation and lipo-apoptosis through PPARα up-regulation and Farnesoid-X Activated Receptor (FXR) activation [122]. In dietary obese mice and human HepG2 treated with fatty acids, miR-21 expression was increased. Furthermore, several studies pointed out a strong miR-21 up-regulation in activated HSCs and this miRNA has been also proposed as biomarker for myocardial fibrosis [122,123,124]. miR-21 may modulate fibrogenesis by targeting phosphatase and tensin homolog (PTEN) thus favoring Akt activation [123]. Additionally, ablation of miR-21 blocks epithelial-mesenchymal transition (EMT) of hepatocytes and suppresses Extracellular signal-regulated kinase 1 (*ERK1*) during HSCs activation through regulation of both *HNF4α* and MAPK inhibitor Sprouty 2 (*SPRY2*) expression [121]. Recently, miR-21 has been proposed to play a role in steatosis progression towards HCC by inhibiting HMG-Box Transcription Factor 1 (*HBP1*). HBP1 enhanced p53 activity which usually suppresses cell cycle-related proteins and lipogenesis by blocking Central Communication Network (CCN) D1/B1 and SREPB1c, respectively [125].

### 6.4. miR-221/222

miR-221 and its homologous miR-222 are a cluster of ~22 nucleotide which are encoded on the X chromosome from a single transcript and differ for only 3 nucleotides downstream the seed region influencing miRNA activity [126] . Although they were widely studied in the oncologic field, Ogawa et al. reported the first evidence that miR-221 and miR-222 were up-regulated in 26 patients affected by NASH in a fibrosis-dependent manner and correlated with *Col1A* and *αSMA* expression in activated primary mouse HSCs. Increased levels of miR-221/222 were also observed in mice with hepatic fibrosis induced by thioacetamide (TAA) administration or MCD diet compared to controls [127].

As mentioned, miR-221/222 gained a great interest in cancer research. Pineau et al. find out a panel of 12 miRNAs which may define progressive liver disease. According to the type of cancer biology, miR-221/222 could function either as tumor suppressor or oncogene (oncomiRs). In the liver, miR-221 seems to favor tumorigenesis by targeting tumor suppressor p27 or, alternatively, DNA damage-inducible transcript 4 (DDIT4), an essential regulator of mTOR kinase activity [116]. Antimirs-based strategies revealed that miR-221 suppression prevented HepG2 cell growth, invasion and induced apoptosis. Stable over-expression of antimir-221 in subcutaneously implanted HepG2 in nude mice prevented tumor establishment and organ metastasis [128].

In addition, hepatic miR-221 over-expression was correlated with poor prognosis in patients stratified for HCC staging [129].

**Figure 1:**
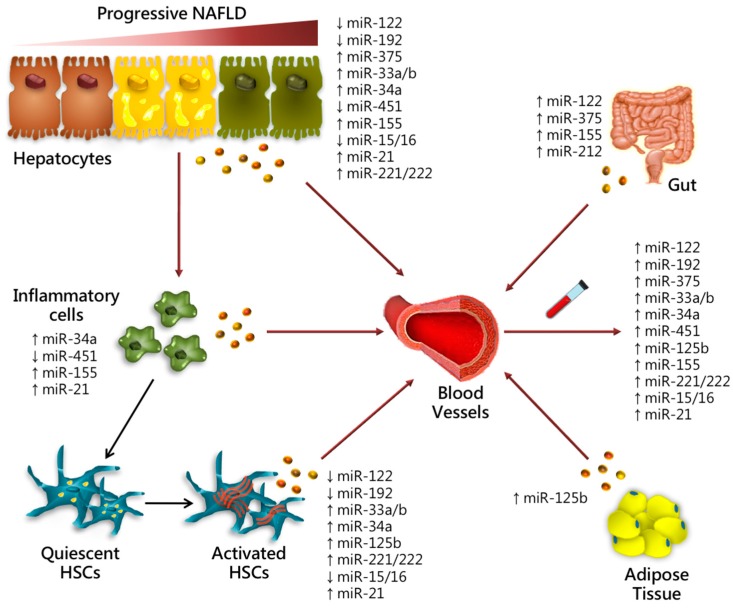
Role of miRNAs in progressive NAFLD. Schematic illustration of candidate miRNAs demonstrated to affect the progression of liver damage. In this figure, we reported the relative miRNA expression in different hepatic cell types (hepatocytes, inflammatory cells i.e., Kupffer cells, HSCs) and tissues (gut and VAT) influencing early and late stages of liver injury. Several miRNAs can be secreted into the circulation triggering steatosis onset, inflammation and ECM deposition. Circulating miRNA can be released via passive (i.e., cell death in apoptotic bodies) or active processes inside exosomes, micro vesicles, HDL and AGO2 and can be easily detected in blood circulation as diagnostic, prognostic and predictive biomarkers.

miRNAs which are involved in progressive NAFLD in different cell type, representing possible candidate biomarkers, are listed in Figure 1 and Table 1.

## 7. miRNA Hereditability in NAFLD

The major epidemiological modifiers in the epidemiology of NAFLD are obesity, T2DM and dyslipidemia [130]. Sedentary lifestyle and high caloric diet consumption are the main environmental factors that contribute to NAFLD. Nevertheless, NAFLD has also a strong heritable component [131,132]. Previous studies have been demonstrated that serum biomarkers (e.g., γ glutamyl transferase, GGT) are hereditable [133]. Hereditability of epigenetic factors is not completely understood. Recent studies in mammals have provided evidence that exposure to environmental stressors can drive stably inherited phenotypic adaptations in offspring that are inherited by epigenetic, rather than genetic mechanisms and some of these studies concern the development of liver disease. Indeed, male inbred mice fed a low protein diet had offspring that increased liver expression of genes involved in lipid and cholesterol metabolism [134]. Previous studies of DNA methylation in monozygotic twins showed a great hereditability with similar epigenetic profiles between twins [135] although the main differences in epigenetic variability were observed in twins who differed in lifestyle and age. Recently, Zarrinpar et al. by using a twin-study design, demonstrated that serum miRNAs explain discordancy between twins with and without NAFLD. In particular, miR-331-3p and miR-30c not only explained NAFLD discordancy between twins but also were significantly different between participants with and without NAFLD. Participants of this study although genetically similar lived separately as adults and environment was not a confounding factor. miR-122 which has been associated with NAFLD, seems not to be hereditable. It could be speculated that miR-122 could be the result of environmental perturbations, and hence, not linked to host genetics, whereas miR-331-3p and miR-30c could be more affected by genetic processes [136].

## 8. Epigenetic Therapies Which Target miRNAs

A translational epigenetic therapy in liver disease is represented by the miR-122 antagonist miravirsen (SPC3649), a locked nucleic acid-modified DNA phosphonothioate antisense oligonucleotide that sequesters miR-122 in a stable heteroduplex, inhibiting its function. Preclinical studies showed that miR-122 is essential for stability and propagation of HCV RNA [138]. Miravirsen was the first parenterally administered miRNA drug developed against HCV with a potential impact on NAFLD since many genes regulated by miR-122 are involved in lipid metabolism. Studies in chimpanzees showed a marked suppression of plasma and liver HCV RNA in animals that received the highest dose of miravirsen [139] whereas dose-dependent reductions in HCV RNA levels were observed after 5 weekly administrations of miraversen in non-cirrhotic patients infected with HCV genotype 1 without evidence of viral resistance [140]. However, other miR-122 targets could be affected by miravirsen as demonstrated by phase IIa study results which revealed that antagonism lowered serum cholesterol levels. In general, therapeutic approaches based on miRNA targeting may be problematic due to their high redundancy and multi-functionality. Further studies will be necessary to explore the applicability of miRNA modulators in the therapy of NAFLD.

Although there are no approved studies for the therapeutic use of miRNAs in HCC, MRX34, a liposome-formulated miR-34 mimic developed by Mirna Therapeutics (Austin, TX, USA), produced complete HCC regression in mouse models [141] and a phase I study is currently recruiting patients with advanced liver cancer for HCC therapeutic intervention (NCT01829971).

It has been described, both in vivo and in vitro, the efficacy of miRNAs to increase the response to Sorafenib in HCC. Sorafenib, a potent multikinases inhibitor, represents the ongoing therapy approved for the management of advanced HCC by hampering tumor cell proliferation and angiogenesis. To date, there are just few evidence of the prognostic role of aberrant serum miRNAs predicting Sorafenib response [137,142]. It has been described that miR-221 modulates Sorafenib resistance through inhibition of caspase-3-mediated apoptosis [137]. Also, miR-21 participates to the acquired resistance to Sorafenib by suppressing autophagy through the Akt/PTEN pathway. Anti-miR-21 oligonucleotides tested in animal models significantly reduced tumor size by 51.5%, and combination therapy with Sorafenib resulted in a further reduction of tumor size by 74.5% [143]. Tang et al. generated an artificial long non-coding RNA (AlncRNA), which simultaneously targets and inhibits multiple miRNAs including miR-21, miR-153, miR-216a, miR-217, miR-494 and miR-10a-5p, which have been shown to be up-regulated in Sorafenib-resistant cells and participate in the mechanisms underlying Sorafenib resistance. Ad5-AlncRNA inhibited proliferation and induced apoptosis of Sorafenib-resistant cells and enhanced the effects of Sorafenib in vitro and in animal models by blocking autophagy [144]. In another study, intra-tumor injection of miRNA-122 packaged in exosomes significantly increased the antitumor efficacy of Sorafenib on HCC in vivo [145]. Finally, rhamnetin, a flavonoid from sea buckthorn, acts as a promising sensitizer of Sorafenib and overcomes multidrug resistance in HCC by regulating miR-34 and NOTCH-1 expression [146]. Relying on the results obtained in vivo and in vitro, Regulus Therapeutics (San Diego, CA, USA) is developing anti-miR122 (RG-101) and anti-miR103 (RG-125) for anti-HCV and anti-NASH, respectively. Enrollment of patients in phase II has been completed for anti-miR122 and enrollment of patients in phase I is about to start for anti-miR103 with AstraZeneca (London, UK) [147].

In summary, the evaluation of potential biomarkers predicting the response to anti-tumoral treatments is a huge challenge for clinical personalized interventions. Thus, analysis of circulating miRNAs may be a valuable strategy for the diagnosis of HCC and prediction of survival of patients receiving Sorafenib treatment.

It is worth discussing whether the reported changes in miRNAs are reversible upon pharmacological treatments of NASH. For instance, it has been described that miR-21 plays an important role in NASH pathogenesis by inhibiting PPARα. Rodrigues et al. found that miR-21 ablation in a mouse model of NASH reduced hepatic steatosis, inflammation and fibrosis and these effects were partly attributable to PPARα. Notably, antagomiR-21 combined with OCA administration, which activates FXR, inhibited NASH development suggesting that this combination could represent an interesting therapeutic strategy for the treatment of NASH [122]. Additionally, chenodeoxycholic acid (CDCA) therapy seems to modulate the expression of the miRNAome in primary human hepatocytes. Krattinger et al. showed that CDCA influences the expression of distinct miRNAs that regulate the expression of genes implicated in bile and lipid metabolism. In particular, miR-34a was suppressed by CDCA and inversely correlated with key genes involved in bile acid homeostasis [148]. Furthermore, changes in miRNA profile associated with metformin treatment occur in mice fed MCD. In this model of NASH, the expression of several miRNAs including miR-34a was up-regulated whereas that of miR-122, miR-101b, miR-194 was decreased upon metformin exposure [149]. These results suggest that miRNA profile represents a potential tool which may help to clarify the mechanisms underlying the metformin-mediated improvement of hepatic steatosis and fibrosis.

## 9. Single Nucleotide Polymorphism Can Modify miRNA Target Sites

SNPs associated with polygenetic disorders, such as NAFLD, can destroy or create miRNA binding sites. The *PNPLA3* isoleucine to methionine substitution at position 148 (rs738409 C>G encoding for PNPLA3 I148M) is the most robust and well replicated genetic variant associated with NAFLD pathogenesis and progression [7,150].

It has been speculated that disruption of miRNA target binding by *PNPLA3* I148M allele may play a role and partially explain the effect of this gene variant on fatty liver susceptibility. In silico prediction analysis revealed that rs738409 G and C alleles show potentially different miRNA binding sites, suggesting a putative different role in gene regulation. In particular, two miRNAs (hsa-miR-769-3p and hsa-miR-516a-3p) have been predicted to potentially interact in the 3’UTR region of *PNPLA3* gene [151].

In addition, it has been described that *PNPLA3* gene lies between two CCCTC-binding factors-bound sites that could be tested for insulator activity, and an intronic histone 3 lysine 4 trimethylation peak predicts an enhancer element, suggesting an epigenetic modulation of this gene.

Suresh et al. catalogued the SNPs in selected miRNA genes directly or indirectly related to the Warburg effect through a computational analysis. These SNPs can affect regulation of miRNA biogenesis and alter miRNA levels, thereby affecting genes that control aerobic glycolysis, providing useful information for application in cancer therapy [152].

Finally, it has been described that a putative binding site for miR-491-5p resides in 3’UTR of *MMP9*, and the genetic variant (rs1056628 A>C) which is present in this region abrogates its post-transcriptional regulation affecting gastric cancer susceptibility [153].

## 10. Concluding Remarks

Genetics, environment and dietetic habits play a key role in NAFLD development and progression. Epigenetics changes interact with genetic risk factors thus increasing individual susceptibility to NAFLD and possibly explaining the huge phenotypic variability. To date there are not approved drugs for NAFLD and liver biopsy remains the gold standard procedure to diagnose its progressive forms. In this context, there is a growing need to identify novel and trustworthy biomarkers, attempting to distinguish NASH from simple steatosis and to diagnose fibrosis, thus circulating miRNAs have become appealing molecule candidates for diagnosis and staging of liver diseases. Moreover, it has been demonstrated although not in patients that changes in miRNAs are reversible upon treatment with drugs used as therapies for NASH.

The possibility to assign a specific spectrum of miRNA expression to each stage of NAFLD and to formulate panels of miRNAs as suitable molecular biomarkers by combining them with the genetic profile, represents a fundamental innovative breakthrough for identifying new important diagnostic tools to tailor personalized efficient therapeutic interventions and more accurate prognosis, although the issue requires further validations.

## Figures and Tables

**Table 1:** List of candidate miRNAs proposed as non-invasive biomarkers, predictors and therapeutic targets in nonalcoholic fatty liver disease (NAFLD).

Candidate Biomarkers	Serum	Liver	Function	Experimental Model	NAFLD Severity	Ref.
miR-122	Up-regulated	Down-regulated	Lipid metabolism Intestinal permeability modulation Inflammation Fibrogenesis Proliferation	Human MCD mice In vitro	Steatosis NASH Fibrosis HCC	[29,38,39,40,71]
miR-192	Up-regulated	Down-regulated	HSCs activation	Human	Steatosis NASH Fibrosis	[29,54]
miR-375	Up-regulated	Up-regulated	Glucose homeostasis Intestinal permeability modulation Inflammation	Human HFD mice In vitro	Steatosis NASH HCC	[29,55,56]
miR-125b	Up-regulated	Up-regulated	Lipid and glucose homeostasis Adipocytes differentiation HSCs activation Fibrogenesis	Human	Steatosis NASH Fibrosis	[29,87]
miR-33a/b	Up-regulated	Up-regulated	Lipid and cholesterol metabolism Glucose homeostasis HSCs activation	Human Non-human primates HFD mice MCD mice In vitro	Steatosis NASH Fibrosis	[66,69,70]
miR-34a	Up-regulated	Up-regulated	Lipid metabolism Oxidative stress Apoptosis Proliferation	Human HFD mice In vitro	Steatosis NASH Fibrosis HCC	[71,72,75,76]
miR-451	Up-regulated	Down-regulated	Inflammation	Human HFD mice In vitro	NASH	[39,79]
miR-155	Up-regulated	Up-regulated	Lipid metabolism Intestinal permeability modulation Inflammation	Human MCD mice In vitro	NASH	[81]
miR-221/222	Up-regulated	Up-regulated	HSCs activation Fibrogenesis Sorafenib resistance	Human TAA mice MCD mice In vitro	NASH Fibrosis HCC	[116,127,137]
miR-15/16	Up-regulated	Down-regulated	HSCs activation Proliferation and Metastasis	Human CCl4 rats In vitro	Fibrosis HCC	[45,108,109]
miR-21	Up-regulated	Up-regulated	Gut microbiota modulation Inflammation EMT Proliferation	Human HFD mice In vitro	NASH Fibrosis HCC	[92,93,122,125]

## Abbreviations

NAFLD	Nonalcoholic Fatty Liver Disease
NASH	Nonalcoholic Steatohepatitis
HCC	Hepatocellular Carcinoma
HBV	Viral Hepatitis B
HCV	Viral Hepatitis C
T2DM	Type 2 Diabetes Mellitus
VAT	Visceral Adipose Tissue
ALT	Alanine Aminotransferase
AST	Aspartate Aminotransferase
BMI	Body Mass Index
NAS	NAFLD Activity Score
FIB 4	Fibrosis 4
TNM	Tumor, Node and Metastasis
SNP	Single Nucleotide Polymorphism
PNPLA3	Patatin-like phospholipase domain containing-3
TM6SF2	Transmembrane 6 superfamily member 2
MBOAT7/TMC4	Membrane bound O-acyltransferase domain containing 7-Transmembrane channel-like 4
HFD	High Fat Diet
MCD	Methionine Choline-deficient Diet
TAA	Thioacetamide
VLDL	Very Low-Density Lipoprotein
HDL	High Density Lipoprotein
DSS	Dextran Sulfate Sodium
CCl4	Carbon Tetrachloride
UDCA	Ursodeoxycholic Acid
OCA	Obeticholic Acid
FFA	Free Fatty Acid
KCs	Kupffer cells
CDCA	Chenodeoxycholic Acid
HSCs	Hepatic Stellate Cells
ATM	Adipose Tissue Macrophages
IEC	Intestinal Epithelial Cell
ECM	Extracellular Matrix
SREBP	Sterol Regulatory Element-binding Protein
miRNAs	microRNAs
exomiRs	Exosomal miRNAs
LPS	Lipopolysaccharides
TLRs	Toll-like receptors
NF-κB	Nuclear Factor Kappa-Light-Chain-Enhancer of Activated B Cells
PPARα	Peroxisome Proliferator Activated Receptor-alpha
PPARγ	Peroxisome Proliferator Activated Receptor-gamma
PTEN	Phosphatase and Tensin Homolog
ACLS-1	Acyl-CoA Synthetase 1 Long-chain Family Member 1
TGF-β/Smad	Transforming Growth Factor-Beta/Suppressor of Mothers Against Decapentaplegic Miscellaneous
AMPK	AMP-activated Protein Kinase
AdipoR2	Adiponectin Receptor 2
ATG7	Autophagy-related Gene 7
ATG12-5	Autophagy-related Gene 12-5
AEG-1	Astrocyte Elevated Gene-1
YAP1	Yes-associated Protein
SIRT1	Sirtuin 1
HNF4α	Hepatocyte Nuclear Factor 4 Alpha
IL-6	Interleukin 6
IL-8	Interleukin 8
IL-10	Interleukin 10
IL-12p40	p40 Subunit of Interleukin 12
ZO-1	Zonula Occludens-1
UTR	Untranslated region
IL-6/STAT3	Interleukin-6/Signal Transducer and Activator of Transcription 3
HNF6	Hepatocyte Nuclear Factor 6
CK-18	Cytokeratin-18
TNF-α	Tumor Necrosis Factor-alpha
CCL2	C-C Motif Chemokine Ligand 2 (CCL2)
AGO2	Argonaute-2
HIF1-α	Hypoxia-Inducible Factor 1-α
MAPK1	Mitogen-Activated Protein Kinase 1
ABCA1	ATP-binding Cassette Transporter sub-family A member 1
APOA1	Apolipoprotein A1
Col1A1	Type I Collagen A1
αSMA	alpha-smooth muscle actin
PI3k/Akt	Phosphatidylinositol-3-Kinase/ Protein Kinase B
HMGCR	Hydroxymethylglutaryl-CoA Reductase
FGF19	Fibroblast Growth Factor 19
LXRα	Liver X Receptor alpha
FXR	Farnesoid-X Activated Receptor
iNOS	Inducible Nitric oxide synthases
Gα12	Guanine Nucleotide-binding α-subunit 12
GPCRs	G-protein Coupled Receptors
Bcl2	B-cell lymphoma-2
CASP2	Caspase 2
MMP2	Metalloproteinase 2
MMP9	Metalloproteinase 9
HDAC1	Histone deacetylase 1
ERK1	Extracellular Signal-Regulated Kinase 1
SPRY2	Sprouty 2
HBP1	HMG-Box Transcription Factor 1
CCN	Central Communication Network
DDIT4	DNA Damage-Inducible Transcript 4
AlncRNA	Artificial Long Non-coding RNA
DGCR8	DiGeorge critical region 8
GGT	γ Glutamyl Transferase
